# Grammatical number inflection in Arabic-speaking children and young adults with Down syndrome

**DOI:** 10.4102/sajcd.v67i1.702

**Published:** 2020-11-05

**Authors:** Bassil Mashaqba, Haneen Abu Sa’aleek, Anas Huneety, Sabri Al-Shboul

**Affiliations:** 1 Department of English Language and Literature, Faculty of Arts, The Hashemite University, Zarqa, Jordan

**Keywords:** Down syndrome, dual, grammatical number, inflection, Jordanian Arabic, morphology, plural, singular

## Abstract

**Background:**

Individuals with Down syndrome (DS) have more difficulties with the structural aspects of language, including morphology (concatenation and non-concatenation) and syntax (word order and grammatical/concord rules), than with other language components (e.g. vocabulary, phonetics and pragmatics).

**Objectives:**

This study investigates the accuracy of grammatical number inflection produced by Jordanian Arabic-speaking children and young adults with DS. The work also examines the correlation between age and the correct production of singular, dual and plural numbers.

**Methods:**

The study involved 60 monolingual Arabic children and young adults with DS, 30 males and 30 females, enrolled at the Nazik Al Hariri Welfare Centre for Special Education, Amman. The participants were divided into three groups: KG2 (7.1–12.5 years old), school (13.10–17.6) and vocational training (18.3–27.3). The participants’ data were collected from a picture elicitation task and free speech, and the answers were recorded using a smartphone. Tokens were classified into correctly used, incorrectly used or not recognised. Proficiency percentage in using the correct number in correlation with age was calculated adopting Jia’s (2003) composite score of proficiency. The one-way analysis of variance was used to trace the impact of age on the correct performance of number. Post hoc comparisons (guided by the Scheffe test) were calculated for the cumulative results of the scale as a whole to examine the difference in the arithmetic mean between the three groups.

**Results:**

The singular form was the most used by all age groups (83.3%), followed by the plural (27%); the most delayed was dual (10.3%). Intriguingly, the dual form is the most difficult plural pattern because it was the least frequently used pattern in everyday language. Results were in line with other research on morphological markers in individuals with DS (e.g. Penke, 2018). The cumulative results statistically prove the influence of age on the correct use of grammatical number, in favour of the older two groups (total *F* = 29.865, at the level of significance *P* = 0.000), with a higher arithmetic mean of all categories (AM: KG2 = 9.00, school = 15.10, VT = 16.25). Hence, sensitivity to the correct number option increases with age although children and young adults with DS do not reach adult-like performance. The non-recognition cases of the proper number category significantly mark language delay in participants with DS.

**Conclusion:**

The study concluded that inflection for grammatical number is evidently delayed in individuals with DS. Linguistic teaching and training of children with DS (involving families, caregivers and educators) should start from childhood and continue to adulthood to improve their use of dual and plural numbers.

## Background

Down syndrome (DS), also known as trisomy 21, is the most commonly identified genetic disorder, and the most frequent chromosomal defect in live-birth infants as a consequence of non-disjunction, an error in cell division. This error produces an extra copy of chromosome 21, resulting in 47 chromosomes instead of the normal 46. This extra copy of chromosome 21 is the generator of abnormalities and structural and functional anomalies in the body’s systems (Wajuihian, 2016, p. 1). Down syndrome has three types: trisomy 21 (found in 95% of people with DS), translocation (4%) and mosaic (1% – 2%). Only the translocation type is inherited (Wajuihian, 2016, p. 6).

Language is one of the common developmental disabilities in individuals with DS. It is referred to as *delayed* instead of *deficient* ability as people with DS produce their first utterances or words later than their peers. Such delay in language production is also reflected in the more predominant use of gestures by DS children than typically developing children (TDC) (Kim & Ko, 2002). People with DS follow the same developmental stages of language acquisition, although these are rarely complete (Rondal, 2009, p. 138). This incompleteness of language development results from cognitive impairment in the brain that is linked to intellectual disability (Rondal, 2009, p. 138).

Despite the fact that individuals with DS vary in cognitive abilities, they exhibit the same linguistic profile of communication strengths and difficulties (Martin, Klusek, Estigarribia, & Roberts, 2009). Morphology and syntax are the most problematic aspects in DS children, although this improves with age. The major morphological difficulties lie in: ‘producing the proper inflections for nominal number concord as well as the markings for person, number, and tense or aspect on verbs’ (Rondal, 2010, p. 11). In addition, verb patterns and auxiliaries are learnt at a later stage and tend to remain unstable in individuals with DS.

A good deal of literature concentrates on morphological and grammatical acquisition in children with DS, as one of the impaired cognitive domains in DS. The studies conclude that children with DS acquire these patterns at a later stage than typically developing individuals (e.g. Penke, 2018). Language delay is also accompanied by inconsistency and atypical asynchrony in using free morphemes, grammatical morphemes, verb inflections (for person, number and gender), nominal inflection and word use (within noun as well as verb phrases); individual and age differences are also evident (Fabbretti, Pizzuto, Vicari, & Volterra, 1997; Rondal, 2010). Children with DS struggle with functional patterns across languages. English-speaking children with DS tend to delete function words (e.g. pronouns, prepositions, copula and auxiliaries) and bound morphemes (Arias-Trejo & Barrón-Martínez, 2017; Chapman, Seung, Schwartz, & Bird, 1998); Spanish-speaking children use gender, number and tense agreement inconsistently (Lázaro, Garayzabal, & Moraleda, 2014), and German-speaking children omit verbal agreement marking (Penke, 2018).

Whilst several studies have been conducted on children with DS in several languages, no single work has considered the use of grammatical number by individuals with DS in Arabic. Number differs amongst different language systems. For example, English is a concatenative language, whereas Arabic, which belongs to the Semitic family, is non-concatenative. For instance, the number system does not follow a linear process (suffixation) to an extent and irregular plural patterns must usually be experienced and memorised (lexicalised) (for details on the plural system, refer to the next section: ‘Number in Jordanian Arabic’). This complexity may create more language difficulties in Arabic-speaking individuals with DS.

Following the recommendation of Galeote, Soto, Sebastián, Checa and Sánchez-Palacios (2014), more experiments should be carried out in other languages, to examine the role of different language aspects in setting up specific developmental profiles. Arabic is one of the languages that lack research examining the linguistic profile of children and young adults with DS. This study aims to fill in a gap in literature by addressing grammatical number in Arabic, as produced by children and young adults with DS in Jordan. It also identifies differences in this morphological aspect occurring as a result of variables such as age, as a warning sign to conduct continuous language training. A set of clinical and pedagogical implications are suggested to be considered by teachers and therapists for better improvement of individuals with DS. Such implications can be pedagogical because number is not targeted adequately in the process of teaching, especially as Arabic has a complex number morphology which may cause misunderstanding(s). Hence, creative teaching methods should be refined to help individuals with DS express their needs and thoughts. The linguistic behaviour of individuals with DS is highly inconsistent and unclear and characterised by severe delayed use. This study contributes to building, with other studies from different languages, a comprehensive linguistic profile for individuals with DS. The contribution of this work is of paramount importance because no validated and standardised tool currently exists to measure the progress of individuals with DS in Jordanian Arabic (JA). Thus, this work stands in support of recommendations for building such a tool and identifies a set of pedagogical implications that help to find comprehensive intervention.

## Number in Jordanian Arabic

Grammatical number in Jordanian Arabic (JA) is trifold: singular, dual and plural. The singular number is unmarked and represents the word root/stem. The dual number is marked by using the suffix – (t)*e:n* to express all cases, with phonological modification of the suffix from *a:n*/*ajn* or using the plural form (see Table 1). Plural number is divided into two categories: (regular) sound plural (SP) and (irregular) broken plural (BP). Sound plural involves linear by adding suffixes to the singular base without any internal change: stem + -*i:n* for masculine sound plural (MSP; Table 1, data in a) and stem + -*a:t* for feminine sound plural (FSP; Table 1, data in b). Broken plural is formed via non-linear processes, which involve stem re-modification and/or internal vowel change (Table 1, data in c; Mashaqba, 2015; Mashaqba, Al-Khawaldeh, Ghweri, & AlOdwan, 2020; Mashaqba & Huneety, 2017).

**TABLE 1:** Simplified number system in Jordanian Arabic.

Singular	Gloss	Dual	BP	MSP	FSP
*mʕallim*	teacher (m)	*mʕallm-e:n*	*-*	*mʕalm-i:n*	*-*
*mʕallma*(*t*)	teacher (f)	*mʕallimt-e:n*	*-*	*-*	*mʕalm-a:t*
*ʃubba:k*	Window	*ʃubba:k-e:n*	*ʃababi:k*	-	-

BP, broken plural; MSP, masculine sound plural; FSP, feminine sound plural.

Note: The adjectives ‘sound’ and ‘broken’ in no way refer to the linguistic abilities of the children, but rather describe a terminology that is purely Arabic.

In terms of number acquisition in Arabic by typically developing children, the singular form is mastered first, followed by dual number and then the plural (e.g. Aljenaie, Abdalla, & Farghal, 2011; Moawad, 2006). As BP does not follow linear formation rules, it can only be acquired through lexicalisation and learning. It is therefore expected to be mastered after SP (Abdalla, Aljenaie, & Mahfoudhi, 2012).

Age and the type of plural category significantly affect the progress of acquiring the plural system in JA. Sensitivity to the non-concatenative option increases with age and conforms to the three-phase representation of the plural development: ‘pre-morphology, proto-morphology and morphology proper’ (Albirini, 2015, p. 754). The pre-morphological phase is manifested predominantly in 3-year-olds who apply uninflected singular stems and produce some inflected plural forms. This is because the plural system may not be adequately developed. At this stage, no dominant default pattern has yet been formed. This is revealed by equal performance of different plural categories. The proto-morphological stage probably begins at the 4-year stage, where children accurately employ singular uninflected stems accompanied by the excessive use the FSP *–a:t* to any other stems. At the age of 5 and 6 years, children clearly develop their plural system, internalising and increasing the pluralisation through a variety of morphemes. However, the MSP and BP forms remain susceptible to be used mistakenly because of incomplete knowledge of these forms; children still make mistakes as they are selectively asked to apply human and non-human nouns. The morphology proper stage, starting when children are 7 and 8 years old, implies increasing ability in using plural forms. At this stage, children approximate adult-like usage and show better performance of FSP, MSP and BP. This stage involves the over-regularisation strategy that occurs throughout language development (Albirini, 2015).

## Methods

### Participants

The sample of this study was 60 monolingual JA-speaking individuals diagnosed with DS: 10 males and 10 females in each of the three groups. All participants had trisomy 21 type, and they were seen at Nazik Al Hariri Welfare Centre for Special Education in Amman. Data were taken from three age groups: the KG2 group had a chronological age (CA) of 7.1 to 12.5 years (*M* = 9.7, *SD* = 1.86), a mental age (MA) of 3.3 to 5.9 years (*M* = 4.5, *SD* = 0.98) and a mean average IQ = 49.1; the school group’s CA was from 13.10 to 17.6 years (*M* = 15.25, *SD* = 1.38), their MA was 7.2–10.8 years (*M* = 7.95, *SD* = 1.3), and mean average IQ was 55.8; and the vocational training group’s (VT) CA was from 18.3 to 27.3 years (*M* = 22.6, *SD* = 3.94), MA 9.1–16.9 (*M* = 12.7, *SD* = 3.36), and mean average IQ was 56.6. Age was examined because it impacts the use of grammatical number by individuals with DS. IQ scores were determined using the Stanford–Binet IQ test. All participants lived with their families. Based on their clinical records, they had no history of hearing or vision problems, additional medical or behavioural conditions (such as anxiety disorder, psychosis and epilepsy). All participants in this research commonly produced more than two-word utterances in spontaneous speech. This information was confirmed by a speech pathologist and his or her teachers.

### Data collection

To date, research on individuals with DS in JA has no validated and standardised measurement tool. Data were elicited via a picture naming task where each participant was shown 10 pictures on Microsoft PowerPoint 2016, with each picture shown separately for 20 s. All tasks took place in the familiar setting of a comfortable room and involved one participant and the examiner (the second author) in each session. The PowerPoint show involved 10 common items under examination where the order of presentation was the same for all participants and all items. Each task was preceded by a familiarisation practice with two pictures of common objects, which are not part of the 10 items on the PPT; the examiner asked the participant to describe what she or he saw in the picture. In this phase, the examiner gave positive feedback plus the target sentence *naʕam saḥi:ḥ ʃaṭṭu:r* ‘yes you are right, excellent!’, whereas in the experiment itself, no positive feedback was provided. The examiner just said *xalli:-na n-ʃu:f iṣ-ṣu:ra illi baʕd-ha* ‘let’s see the next picture’, and the examiner asked the participant to describe what she or he saw in the picture. Every item was presented in the three cases of the grammatical category of number in Arabic: singular, dual and plural. For the plural number, only the FSP and BPs were tested because they are the default plural patterns. Stating that BP is a (minor) default pattern does not contradict the notion that it is one of the most complex plural forms in Arabic. Considering the lexicon as a whole, BP is non-exceptional (Al-Shboul, Huneety, Mashaqba, Zuraiq, & Al-Omari, 2020; Mashaqba, 2015; Mashaqba et al., 2020; McCarthy & Prince, 1990). Corpus studies on pluralisation system in Arabic confirm that BP is frequent and, to an extent, predictable by native Arabic-speaking adults (Boudelaa & Gaskell, 2002). Although FSP is the default plural, it is concatenated to a limited set of nominal stems; by way of contrast, BP is more frequently used than SP by native Arabic-speaking adults. To elicit the required data, the examiner asked in Arabic: *ʃu: ʃa:jif biṣ-ṣu:ra* ‘What do you see in the picture?’. The first two authors recorded monologues (free speech) uttered by the participants, and conversations between the participants themselves and between them and their teachers. The researchers did not depend on free speech as a method of collecting data from the participants. However, there was a persistent need to make some free conversations with some participants to encourage them (especially the youngest group/KG2) to speak freely as some of them were shy and silent and to notice their production of plural structures naturally when speaking freely. The results of the free speech were not calculated in the statistical analysis. Only the data generated from elicited pictures were counted. All picture naming tasks, monologues and conversations were recorded using a smartphone and transcribed using the acceptable phonetic symbols for Arabic (http://jss.oxfordjournals.org/). To achieve reliable testing and avoid researcher bias, the transcription was double-checked by co-authors and a language therapist, and any case of pronunciation disagreement was excluded. Elicited data were transferred to an Excel sheet.

### Procedure of data analysis

All recorded responses were evaluated for each individual; all analysable tokens for number category were extracted and then classified into correctly used, incorrectly used or not recognised by any age group. When the participant did not say anything, the answer was put in a special pattern called *non-recognition*. The tokens were calculated and given scores. Every correct pattern was given one point and every wrong or not recognised pattern zero. Jia’s (2003) composite score of proficiency (Abdalla et al., 2012, p. 153) was adopted to find out proficiency percentage in using the correct patterns in correlation with age for each pattern:

$$\text{Composite Score of Proficiency}=\frac{\text{Total Correct Plural Tokens}}{\text{Total Plural Contexts}}$$ [Eqn 1]

The correct answers are the forms produced by every native JA-speaking adult as reported in literature. In this work, the researchers and one language therapist, who are native speakers of JA, identified the correct forms. The one-way analysis of variance (ANOVA) statistical test was used to trace the impact of age on the correct performance of number. For more accurate validity, post hoc comparisons (guided by the Scheffe test) were calculated for the cumulative results of the scale as a whole and its correlation with age variables to statistically examine the difference in the arithmetic mean between the three groups.

### Ethical consideration

A consent form was prepared, and ethical clearance was obtained from Scientific Research and Research Ethical Approval Committee of the Hashemite University (Number: 1/2019/2020) and permission was granted by Department of English Language & Literature on 22 September 2019. The consent form was signed by parents or caregivers after they were shown the objectives of the study and assured that all ethical issues would be taken seriously. Confidentiality was ensured as the collected data remained private with identification through code names, and the researchers guaranteed that they would delete all the results from their desktops and/or smart phones after conducting the study if the centre and the participants so wished.

## Results

Participants were divided into three age groups to test the influence of age differences and progress in individuals with DS on the production of grammatical number. Table 2 summarises the frequency of patterns produced.

**TABLE 2:** Frequencies of number patterns performed by Down syndrome participants.‡

Number	Pattern	Frequency		
		KG2 (%)	School (%)	VT (%)
Singular	Using the proper singular form	74	94	82
	Not recognising the lexical/content word under examination	12	1	4
	Using incorrect forms (phonetic fragments, plural form, verbs)	14	5	14
Dual	Using the proper dual form	not attested	14	17
	Using the acceptable plural reflex†	9	20	18
	Not recognising the lexical/content word under examination	11	3	2
	Using incorrect forms (singular, phonetic fragments, verbs, word reduplication)	80	63	63
Plural	Using the proper plural form	7	27	47
	Not recognising the lexical/content word under examination at all	10	2	3
	Using incorrect forms (singular, phonetic fragments, dual form, verbs, word reduplication)	83	71	38

KG2, Kindergarten 2; VT, vocational training group.

† , Any correct plural pattern used instead of the dual number;

‡ , Total tokens are 600.

Applying Jia’s (2003) composite score of proficiency, the three DS groups showed different proficiency scores regarding the three categories of number: singular, dual and plural (see Eqn 1). Considering the singular number, the school group was the most proficient in the correct singular form (94%), followed by the VT group (82%), and the KG2 group (74%). In terms of dual number, more progress was reported for the school and VT groups. The VT group produced the (default) dual and acceptable plural in 35% of cases. Next, and very close, the school group scored the second highest correct use (34%). The KG2 group statistically suffered a weak performance using the correct dual inflection (0% for the dual pattern and 9% for the acceptable plural form). The KG2 group had used the plural forms, with only 7% correct responses, as opposed to 27% for the school group and 47% for the VT participants. The finding that some participants did not use any pattern is of interest. Across all number patterns, the non-recognition cases constituted 16% of the cases of the three numbers; the highest percentage was scored by the KG2 group (33%), followed by the school group (9%) and the VT group (6%).

The arithmetic mean (AM) of the three age groups revealed statistically significant differences in accuracy amongst the groups (Table 3). The VT group scored the highest accuracy mean, followed by the school group and the last KG2 group. Therefore, age has an influence on generating differences in producing grammatical number.

**TABLE 3:** Arithmetic mean and standard deviation in the grammatical number as produced by the three Down syndrome age groups.

Age group	Number of participants	AM	SD
KG2	20	9.00	1.97
School	20	15.10	3.12
VT	20	16.25	4.101

**Total**	**60**	**13.45**	**4.48**

AM, Arithmetic means; SD, standard deviation; KG2, Kindergarten 2; VT, vocational training group

One-way ANOVA confirmed significant statistical differences between the three age groups in the responses of the participants by age (Table 4).

**TABLE 4:** Correlation between age and accuracy of grammatical number production.

Variance	Sum of squares	*df*	Mean square	*F*	Significance level *P*
Between groups	607.300	2	303.650	-	-
Within groups	579.550	57	10.168	-	-

**Total**	**1186.850**	**59**	**-**	**29.865**	**0.000**

The results in Table 4 confirm the existence of statistically significant differences at the significance level (a = 0.05) because of the effect of age on number, where the value of (*F*) on this category registers *F* = 29.865, respectively, at the level of significance (*P* = 0.000). The cumulative results prove a statistical significance of the influence of age on grammatical number (total *F* = 29.865).

Over the cumulative results of the scale as a whole and its correlation with age variables, the post hoc comparisons (guided by the Scheffe test) presented statistically significant differences in the arithmetic mean between the KG2 group and the school group and between the members of the KG2 group and the members of the VT group (Table 5). The differences are in favour of the school group and the VT group over the KG2 group with a higher arithmetic mean of all categories on the total scale.

**TABLE 5:** Post hoc comparisons of age variable in grammatical number.

Age group	Arithmetic mean	KG2	School	VT
KG2	9.00	-	-	-
School	15.10	0.000*	-	-
VT	16.25	0.000*	0.526	**-**

KG2, Kindergarten 2; VT, vocational training group.

* , Significance level (α = 0.05).

## Discussion

One major finding of this study is that participants with DS have difficulties and limited knowledge of the proper number inflection. The incorrect patterns used by the three groups reflect the presence of a mental disorder area in people with DS that causes the linguistic behaviour of the incorrect production of linguistic items in contexts other than their obligatory ones, in addition to usage inconsistency of grammatical morphemes (cf. Fabbretti et al., 1997).

All DS groups scored high rates of proficiency in producing the correct singular number. This could be ascribed to the fact that singular number is the default category and the easiest pattern to acquire first (Albirini, 2015; Mashaqba et al., 2020; Mashaqbeh, 2009). All DS groups produced singular as the most frequent incorrect pattern to express dual and plural, which implies a cognitive delay in using the proper dual/plural form. Typically developing Arabic-speaking children master the dual number at the age of 10 years (Moawad, 2006). Hence, reliance on the (default) singular to express the other inflected numbers denotes that children and young adults with DS are still in the pre-morphological phase, corresponding to that of 3-year typical children (Albirini, 2015). This phase reveals language delay in producing the dual and plural numbers by Jordanian children and young adults with DS compared with typically developing children.

The KG2 group represents a significant linguistic behaviour in producing the grammatical number. The abundance in the number of incorrectly produced patterns (such as phonological fragments, verb patterns and word reduplication) probably refers to linguistic instability and inconsistency in this younger group, which confirms the persistent need to expose individuals with DS to linguistic intervention at early ages. This result is in line with Christodoulou (2015) who found articulation problems and morpho-phonological delayed associations related to DS. Consequently, the phonological difficulties influence the morphological abilities, making it difficult for researchers to distinguish words’ phonological boundaries and thus whether the answers are correct or not correct. Utilising verbs and verbal nouns instead of the target words implies that: (1) the acquisition of the verb form is in progress at this age (Chapman et al., 1998). The participants showed limited performance in verbs in their mandatory contexts, and this pattern reveals inconsistency in using grammatical forms in DS (Fabbretti et al., 1997), especially when describing verbs. (2) It is an apparent sign of the influence of the surrounding environment on young individuals with DS, educational centres in particular. The researchers set out with this belief when observing that children pronounce words such as *kita:ba* ‘writing’ in its standard form, which means they have learned it in a formal way. On the other hand, this pattern indicates immature linguistic ability in this young group as a result of cognitive problems and appears in the wrong context in which the pattern is used. (3) By using word reduplication, the participants resemble the children recorded by Mashaqbeh (2009) in the 3–4 year age group who use it only to describe the dual form. Using word reduplication indicates that the KG2 participants probably understand the multiple characteristics of things but have problems in acquaintance with the morphological form.

The participants in the school group were more competent in using the singular number than the younger KG2 group and the older VT group. This linguistic competence in relation to the groups’ age was also found by Fabbretti et al. (1997). In comparison with the VT group, this may correspond to individual abilities found amongst the participants. On the other hand, this finding is incompatible with Chapman et al. (1998) and Rondal and Comblain (2002), who proposed that linguistic change from adolescence into late adulthood is not attested. Remember that the school group outperformed the VT group in other aspects in this study (our data reported instances where the school group is outperformed by the VT group). In sum, it can be claimed that there are signs of language decline in individuals with DS that starts in young adulthood.

Down syndrome groups endure a kind of inability to produce the dual number. Excluding the acceptable plural number, the typical dual form is altered to be the weakest number in DS. This signifies that the dual number is a secondary number category. Also, the deficient use of the real dual number form by the DS participants contradicts the linguistic behaviour of the TDC children who master and better perform the dual number prior to the plural number because it has one single form. Instead, DS children and young adults rely more on their environmental language input, for the plural number form is more used in JA than the dual number form. Hence, the dual number is acquired after the plural in DS, unlike in the typical children reported in Moawad (2006) and Aljenaie et al. (2011), reflecting the sensitivity to the surrounding environment by individuals with DS. Thus, children and young adults with DS perform a considerable amount of dependency in using more social language (Martin et al., 2009). In this sense, but different from Chapman et al. (1998) and Rondal and Comblain (2002), it should be noted that the use of the real dual form becomes better amongst the older groups, associated with less reliance on the plural form, which implies an acquisition-in-progress process for this pattern (see Table 2).

Unlike the findings of Chapman et al. (1998) and Rondal and Comblain (2002), the three age groups show a distinguishable gap in improving proficiency of plural inflection (KG2 = 7%, school = 27%, and VT = 47%), which may be attributed to the significant role of age (Albirini, 2015; Mashaqba et al., 2020). The findings extracted from the one-way ANOVA test reveal a significant statistical variance as well as transcendence in favour of the VT group preponderantly (see Tables 2–4). Both school and VT groups exhibit large-scale variance from the KG2 group. The two groups do not score an extended variance, although the VT group is better. The plural form is, however, considered as a weakness area in DS; it is the second most commonly and correctly used number after the default singular, as it is used to denote the dual number more than the typical dual form and the canonical plural form. This is the opposite of acquisition behaviour in typically developing children (Aljenaie et al., 2011). In addition, it is at variance with the argument that children with DS acquire the linguistic patterns at a similar phase to TDC children.

As age has a significant role on linguistic performance, linguistic training must be available from childhood and continue to adulthood. Families should pay attention to linguistic input for its role on the linguistic performance in individuals with DS. They also need early teaching to recognise the things around them and how to name these things appropriately. The study recommends more concentrated teaching and training given regarding the dual form in the first place and the plural number in the second place, because they contain a variety of inflection constructions and patterns, which pose a difficulty to many individuals with DS. Lastly, older individuals with DS must be given more training because there is the possibility of language decline with age.

The non-recognition pattern involves cases in which the participants kept silent, referred to names of other items, or pointed at different items found in the class (with their fingers or eyes) where the test is conducted. Both silence and body language are attributed to the fact that children with DS rely heavily on non-verbal modes of communication when they are in formal or unfamiliar situations, bearing in mind that they are not accustomed to the researchers (cf. Martin et al., 2009). The high frequency of this pattern reveals the instability and language deficiency in DS. Instability clearly appears in the VT group’s responses, as the participants are not performing to their ideal age competence. The members of the KG2 group register the highest percentage of this pattern where they do not use the items under examination. The non-recognition cases observed along the examination process stress the need for integrating individuals with DS in different societies and new circumstances to learn about the things around them. The settings of the test conducted in this study reveal how acquaintance and familiarity strongly impact language comprehension and production and the psychological status in individuals with DS, especially young groups.

## Conclusion

This work starts from the belief that Arabic is one of the languages that lacks scientific research on language aspects (particularly morphology) of people with DS. In this account, we believe that the study findings sustain and encourage evolution of the educational curriculum concerning the language-learning phase in Arabic-speaking society. The findings conclude that Jordanian children and young adults with DS have problems in producing numbers (singular, dual and plural). The singular number is the most accurately used because it is the unmarked pattern, followed by the plural number. The KG2 group (the youngest group) is distinguishable from the other two groups as it produces patterns that are not used by other groups to describe the singular category, despite its simplicity. The participants show weaknesses in using the real dual pattern, but more reliance on the plural pattern. The non-recognition cases are important signs indicating language delay in DS and how individuals with DS are concerned and responsive to the surrounding circumstances. Finally, age has a significant influence on language behaviour in DS. As a whole, there is a language improvement in the production of number as age increases. Therefore, children with DS need an early language intervention that must continue into adulthood. Based on these conclusions, we recommend further investigations of derivational aspects such as verbal derivatives, denominals and denominatives. Furthermore, because Arabic lacks this kind of study, we suggest more research into other linguistic levels, especially morpho-phonological levels, which are clearly delayed in DS and significantly affect communication with other people. Such investigations might also consider the impact of gender on the language of individuals with DS.
